# A monoclonal antibody-based immunoassay to measure the antibody response against the repeat region of the circumsporozoite protein of *Plasmodium falciparum*

**DOI:** 10.1186/s12936-016-1596-8

**Published:** 2016-11-08

**Authors:** Kristina Radin, Frederic Clement, Erik Jongert, Yann G. J. Sterckx, Christian Ockenhouse, Jason Regules, Franck Lemiale, Geert Leroux-Roels

**Affiliations:** 1 Center for Vaccinology (CEVAC), Ghent University, Ghent, Belgium; 2 GSK Vaccines, Rue de l’Institut 89, B-1330 Rixensart, Belgium; 3 Structural Biology Research Center (SBRC), VIB, Pleinlaan 2, B-1050 Brussels, Belgium; 4 Research Unit for Cellular and Molecular Immunology (CMIM), VUB, Pleinlaan 2, B-1050 Brussels, Belgium; 5 PATH Malaria Vaccine Initiative (MVI), 455 Massachusetts Avenue NW, Washington, DC 20001 USA; 6 Walter Reed Army Institute of Research (WRAIR), 503 Robert Grant Ave., Silver Spring, MD 20910 USA

**Keywords:** Malaria, Competition ELISA, Enzyme-linked immunosorbent assay, Immunoassay, Circumsporozoite protein

## Abstract

**Background:**

The malaria vaccine candidate RTS,S/AS01 (GSK Vaccines) induces high IgG concentration against the circumsporozoite protein (CSP) of *Plasmodium falciparum*. In human vaccine recipients circulating anti-CSP antibody concentrations are associated with protection against infection but appear not to be the correlate of protection. However, in a humanized mouse model of malaria infection prophylactic administration of a human monoclonal antibody (MAL1C), derived from a RTS,S/AS01-immunized volunteer, directed against the CSP repeat region, conveyed full protection in a dose-dependent manner suggesting that antibodies alone are able to prevent *P. falciparum* infection when present in sufficiently high concentrations. A competition ELISA was developed to measure the presence of MAL1C-like antibodies in polyclonal sera from RTS,S/AS01 vaccine recipients and study their possible contribution to protection against infection.

**Results:**

MAL1C-like antibodies present in polyclonal vaccine-induced sera were evaluated for their ability to compete with biotinylated monoclonal antibody MAL1C for binding sites on the capture antigen consisting of the recombinant protein encompassing 32 NANP repeats of CSP (R32LR). Serum samples were taken at different time points from participants in two RTS,S/AS01 vaccine studies (NCT01366534 and NCT01857869). Vaccine-induced protection status of the study participants was determined based on the outcome of experimental challenge with infected mosquito bites after vaccination. Optimal conditions were established to reliably detect MAL1C-like antibodies in polyclonal sera. Polyclonal anti-CSP antibodies and MAL1C-like antibody content were measured in 276 serum samples from RTS,S/AS01 vaccine recipients using the standard ELISA and MAL-1C competition ELISA, respectively. A strong correlation was observed between the results from these assays. However, no correlation was found between the results of either assay and protection against infection.

**Conclusions:**

The competition ELISA to measure MAL1C-like antibodies in polyclonal sera from RTS,S/AS01 vaccine recipients was robust and reliable but did not reveal the elusive correlate of protection.

**Electronic supplementary material:**

The online version of this article (doi:10.1186/s12936-016-1596-8) contains supplementary material, which is available to authorized users.

## Background

Malaria caused by *Plasmodium falciparum* infection remains a major cause of morbidity and mortality worldwide. In 2015, 214 million clinical malaria cases resulted in an estimated 438,000 deaths, mostly in children and pregnant women in sub-Saharan Africa [1]. Over the past decades significant efforts have been made to develop a malaria vaccine but this process is hampered by the ability of *Plasmodium* species to evade and suppress the host immune response [2, 3] and by the incomplete understanding of how protective immunity to malaria develops [4–7].

Several vaccine candidates, targeting different stages of the parasite life cycle have been developed and shown varying degrees of success upon evaluation [8, 9]. The most advanced malaria vaccine candidate directed against *P. falciparum* is RTS,S/AS01 (GSK Vaccines). This vaccine targets the pre-erythrocytic stage of the parasite and focuses on the circumsporozoite protein (CSP). It consists of 19 NANP amino acid repeat units followed by the complete C-terminal domain without the GPI anchor of the CSP fused to the hepatitis B surface antigen (HBsAg) [10]. Efficacy trials have shown that over the first 18 months following three doses of RTS,S/AS01, malaria cases were reduced by almost half in children aged 5–17 months at the time of first vaccination and by 27% in infants aged 6–12 weeks. At study end, four doses of RTS,S/AS01 reduced malaria cases by 39% over 4 years of follow-up in children, and by 27% over three years of follow-up in infants [11, 12]. In July 2015, the Committee for Medicinal Products for Human Use (CHMP) of the European Medicines Agency (EMA) has adopted a positive scientific opinion for the RTS,S/AS01 vaccine in children aged 6 weeks to 17 months.

RTS,S/AS01 vaccination induces high IgG concentrations against the NANP repeat region of CSP and moderate to high CD4^+^ Th1 responses against flanking region peptides [13–15]. Both responses are associated with protection, but an exact correlate of protection has not yet been defined. While some studies show no direct association between the anti-NANP IgG concentration and protection against clinical disease [16, 17], others suggest that antibodies play a key role in RTS,S/AS01-mediated protection [13, 18–22]. It has been demonstrated that administration of human monoclonal antibodies (mAbs, called MAL1C, MAL2A, MAL3B) derived from an RTS,S/AS01 vaccine recipient and directed against the NANP repeat region of CSP to immune deficient mice with humanized livers was able to convey protection from infection with *P. falciparum* in a dose-dependent manner [23].

RTS,S/AS01-induced antibodies are quantified with a validated ELISA that uses R32LR recombinant protein as a capture antigen [24]. There is evidence for the protective capacity of RTS,S/AS01-induced antibodies in humans, but the correlation between protection and antibody concentrations is far from being perfect. The dose-dependent protection conveyed by RTS,S/AS01-induced mAbs in the humanized mouse model [23] encouraged us to investigate whether a correlation may exist between the protective capacity of RTS,S/AS01 vaccine-induced polyclonal antibodies and their content of MAL1C-like activity. Therefore a competition assay has been developed to measure MAL1C-like activity of polyclonal, vaccine-induced sera. Sera derived from participants in two RTS,S/AS01 trials were analysed with both the MAL1C-competition ELISA and the validated R32LR ELISA. The results of both assays were compared and correlated with protection status against *P. falciparum* infection of these vaccine recipients following a sporozoite challenge 2 weeks following last vaccine dose.

## Methods

### Serum samples

Serum samples from participants in the two clinical trials were analysed to evaluate the presence of both MAL1C-type activity and R32LR-binding in the reference ELISA.

Study 1 (ClinicalTrials.gov Identifier: NCT01366534) evaluated whether administration of two investigational malaria vaccines (RTS,S/AS01_B_ from GSK Vaccines and Ad35.CS.01, a replication deficient adenovirus type 35 circumsporozoite malaria vaccine from Crucell) combined in one immunization schedule increased protection against malaria infection as compared to protection induced by RTS,S/AS01_B_ alone. A full report of the study has recently been published [25]. Sera from 46 study participants analysed here were taken before the 1st vaccine administration (Day 0), 4 weeks after the 2nd dose (D56) and 3 weeks after the administration of the 3rd vaccine dose, immediately before controlled human malaria parasite infection (CHMI), through the bite of 5 *P. falciparum*-infected *Anopheles stephensi* mosquitoes (Day 77).

Study 2 (ClinicalTrials.gov Identifier: NCT01857869) evaluated safety, immunogenicity, and efficacy of RTS,S/AS01_B_ administered intramuscularly as standard doses at 0–1 month and a 1/5th standard dose at 7 months (delayed fractional dose group, 017) as compared to RTS,S/AS01_B_ administered as three standard doses at monthly intervals (0, 1, 2 months group, 012) in healthy malaria-naïve volunteers aged 18–50 years. A full report of the study has recently been published [26]. Samples analysed in the present study were taken prior to vaccination, 4 weeks after the second dose (D56) and 3 weeks after the 3rd dose (D217) prior to CHMI in the former group and prior to vaccination, prior to the 3rd dose (D196), and 3 weeks after the 3rd dose (D217) prior to CHMI in the latter group.

Study samples were analysed in a blinded fashion. Following analysis, information was obtained about sampling time and subject status (protected/unprotected). Protection is defined as absence of parasitaemia in blood samples 28 days post CHMI.

Negative control serum samples were obtained from healthy adults living in malaria non-endemic areas and considered as anti-CSP repeats antibody seronegative. Positive control samples were pools of post-vaccination serum samples, used in the R32LR ELISA. The low positive control (LPC) and high positive control (HPC) have an antibody content of 9.48 and 99.94 EU/ml, respectively.

### Human monoclonal antibodies

Human monoclonal antibodies (mAb), MAL1C, MAL2A and MAL3B, were generated in 2008 in the Center for Vaccinology (Ghent, Belgium) using peripheral blood mononuclear cells (PBMC) from the individual vaccinated with RTS,S/AS01 who showed the highest anti-R32LR antibody concentration one month after the 3rd vaccine dose (ClinicalTrials.gov Identifier: NCT00443131) [27]. Six days after injection of human PBMC in the spleens of conditioned SCID mice, the spleens were harvested and the cells, a large fraction of which were human B lymphocytes, were fused with K6H6/B5 heteromyeloma cells, and grown in hybridoma selection medium as described [28]. Hybridoma culture supernatants were tested for the presence of human anti-R32LR antibodies and cultures producing anti-R32LR antibodies were selected, expanded and subcloned. The mAbs MAL1C, MAL2A and MAL3B are of the IgG1 isotype and directed against the repeat region of circumsporozoite protein (CSP).

### Biotin labelling of monoclonal antibody MAL1C

Biotin labelling of human monoclonal antibody MAL1C was performed using the EZ-Link™ Sulfo-NHS-LC-Biotinylation Kit (Thermo Scientific) according to the manufacturers guidelines. Briefly, a 20-fold molar excess of Sulfo-NHS-LC-Biotin was added to MAL1C solution in phosphate buffer saline (PBS) and the reaction was incubated on ice for 2 h. After the reaction, excess biotin reagent was removed using a desalting column provided with the kit. The degree of biotin labelling was determined with the HABA (4′-hydroxyazobenzene-2-carboxylic acid)/avidin solution provided with the kit and following the instruction manual. Biotinylated MAL1C (B-MAL1C) was diluted with PBS and aliquots were stored at −20 °C. Once thawed the B-MAL1C was stored at 4 °C. Specificity of the biotin labelled MAL1C was tested with the R32LR ELISA [24]. To examine the impact of biotinylation on the functional integrity of MAL1C, the antibody concentration was measured using both the standard R32LR ELISA (see below) and a human IgG ELISA Quantitation Set (Bethyl Laboratories, Montgomery, TX, USA). The latter was performed following the manufacturer’s guidelines.

### Capturing antigens in ELISA

R32LR protein is a recombinant repeat region of the *P. falciparum* circumsporozoite protein produced in AR58 *Escherichia coli* strain, as already described [29]. His-R32LR was constructed with six histidine residues at the N-terminus and produced in the BLR(DE3) *E. coli* strain as described in [24]. After purification, both antigens were kept in 0.2 M phosphate buffer, pH 6.5 and stored in aliquots at −80 °C.

### R32LR ELISA

The R32LR ELISA was performed as described previously [24]. Briefly, antigen R32LR was coated onto a 96-well polystyrene plates (F96, MaxiSorp, Nunc). After coating, plates were washed three times with phosphate-buffered saline (PBS, pH 7.4) supplemented with 0.1% Tween-20 (v/v; Sigma, ref P1379). After blocking the plates with blocking buffer, consisting of PBS (pH 7.4) containing 0.1% Tween-20 (v/v) and 0.5% skimmed milk (Becton–Dickinson, ref 232100), for 2 h at room temperature (RT) on an orbital shaker, serial dilutions of serum samples were added to the plates and incubated for 2 h at 37 °C. The plates were washed and polyclonal rabbit anti-human IgG/HRP was added. After a final washing step the chromogen substrate [3,3′,5,5′-tetramethylbenzidine solution (Sigma, ref T-0440)] was added and incubated for 15 min at RT on an orbital shaker. The reaction was stopped by the addition of 100 μl of 1 N sulphuric acid, before reading the assay plate at 450 nm in a microtiter plate reader. The titers were calculated from a standard curve with the software SoftmaxPro (using a four parameters equation) and expressed as EU/ml. The cut-off for the anti-CSP ELISA was 0.5 EU/ml.

### Surface plasmon resonance

Surface plasmon resonance experiments were performed on a BIAcore T200 system (GE Healthcare). The interactions between the CSP-derived peptides ([NANP]_6_ and R32LR) and the mAbs (MAL1C, MAL2A, MAL3B) were analysed on a CM5 chip. The mAbs were immobilized using the following procedure. Using a flow rate of 5 μl/min the carboxylated dextran matrix was activated by a 7-min injection of a solution containing 0.2 M *N*-ethyl-*N*′-(3-diethylamino) propyl carbodiimide (EDC) and 0.05 M *N*-hydroxysuccinimide (NHS). For the covalent coupling, the mAbs were prepared at a concentration of 10 μg/ml in coupling buffer (50 mM sodium acetate pH 5.0) and were subsequently injected until about 2500 resonance units (R.U.) was immobilized. The surface immobilization was then blocked by a 7-min injection of 1 M ethanolamine hydrochloride. The reference surface was treated only with EDC, NHS, and ethanolamine. Data for the interaction between the CSP-derived peptides and the mAbs were collected in the format of a kinetic titration [30]. Sensorgrams for the CSP-derived peptides were collected at five different concentrations plus a 0 concentration (injection of running buffer) at a flow rate of 30 μl/min and a data collection rate of 10 Hz. Analyte injections were performed with association phases of 180 s and dissociation was allowed to occur for 600 s. Prior to data analysis, reference and zero concentration data were subtracted from the sensorgrams. For kinetic analysis, the data were analysed with a 1:1 Langmuir binding model [30]. In the case of the mAb-[NANP]_6_ interaction, fitting the data with the aforementioned model reports the affinity constant (K_D_) for a 1:1 interaction. However, for the mAb-R32LR interaction, we believe that the mAb- R32LR interaction is the result of an avidity effect because of the relatively long repeat region in this CSP-derived protein. Hence, the affinity constant obtained from fitting the data with a 1:1 interaction model is reported as apparent (K_D,app_).

### MAL1C-competition ELISA

MAL1C-like activity in polyclonal sera was measured as follows. His-R32LR was coated (100 μl/well of a solution of 0.5 μg/ml prepared in 0.05 M carbonate/bicarbonate buffer, pH 9.4-9.8) onto a 96-well polystyrene plate (F96, MaxiSorp, Nunc) for 14–16 h at 5 ± 3 °C. After coating, plates were washed 3 times with phosphate-buffered saline (PBS, pH 7.4) supplemented with 0.1% Tween-20 (v/v; Sigma, ref P1379). Nonspecific binding sites were saturated with 200 μl/well of blocking buffer, consisting of PBS (pH 7.4) containing 0.1% Tween-20 (v/v) and 0.5% skimmed milk (Becton–Dickinson, ref 232100), for 2 h at room temperature (RT) on an orbital shaker. After blocking the plate was turned upside down to remove blocking buffer, and gently tapped down on clean blotting paper. Eight twofold dilutions of controls and samples were made in buffer containing a fixed amount of B-MAL1C. The determination of the optimal amount of B-MAL1C is described in “Results” section. These mixtures were then added to the plate (100 μl/well) and incubated for 2 h at 37 °C. The plates were washed three times before peroxidase-conjugated streptavidin (Streptavidin-HRP, GE Healthcare Life Sciences, RPN4401) diluted in blocking buffer was added for 30 min incubation at RT. The determination of the optimal amount of Streptavididn-HRP is described in “Results” section. After another washing step, the chromogen substrate [3,3′,5,5′-tetramethylbenzidine solution (Sigma, ref T-0440)] was added and incubated for 15 min at RT on an orbital shaker. The reaction was stopped by the addition of 100 μl of 1 N sulphuric acid, before reading the assay plate at 450 nm in a microtiter plate reader. The signal obtained is inversely proportional to the amount of MAL1C-like antibodies present in the samples (competition assay). The amount of antibody competing with MAL1C mAb for binding to the coated R32LR-His is quantified by comparison to a serum not containing anti-CSP antibodies. Results are expressed as the half maximal inhibitory concentration (IC50) that was calculated using the Softmax Pro software (Molecular Devices, Sunnyvale, CA, USA). Individual OD values of 8 dilutions of each sample are converted into % Inhibition (as compared to reference). A four-parameter logistic curve is fitted for each sample.

The Inter-Assay stability was monitored by including a low and high anti-CSP antibody-containing serum on each assay plate. The resulting IC50 of both controls were calculated as outlined above. Low and High anti-CSP containing controls resulted in a mean IC50 value of 11.80 and 27.14, respectively. The observed imprecision (%CV) for both controls were 10.99–16.92%.

In each plate the negative control (identical serum used as calculation reference) was used to evaluate the assay noise and therefore indirectly determine the ability of the assay to discriminate positive results (MAL1C-like antibodies containing samples) from negative sera. Based on 37 observations a technical assay threshold could be set at an IC50 value of 9.92.

### Statistical analysis

Comparisons between groups (e.g. protected versus unprotected) were done with Mann–Whitney U test. The correlation between antibody data measured with R32LR ELISA and MAL1C-competition ELISA were examined using linear regression and Pearson’s correlation analysis.

## Results

### Biotin-labelled MAL1C retains antigen-binding quality

Biotin labelling of MAL1C was performed using the EZ-Link™ Sulfo-NHS-LC-Biotinylation Kit (Thermo Scientific). Using the HABA assay for measuring the level of biotin incorporation that is provided with the EZ-Link™ Sulfo-NHS-LC-Biotinylation Kit the degree of biotin incorporation was determined to be 4:1 (mmol of biotin per mmol of protein). This is within the range that can be achieved with this kit. To examine whether the biotinylation procedure had altered structural and/or antigen-binding properties of MAL1C, the IgG content and the antigen-binding capacity of the B-MAL1C were measured with the IgG ELISA Quantitation Set and the standard R32LR ELISA, respectively. The antibody content of the B-MAL1C was 3.54 mg/ml with the R32LR assay and 3.73 mg/ml with the IgG ELISA, showing that the labelling procedure had not detectably altered the mAb.

### Development of the MAL1C-competition ELISA

**Fig. 1:**
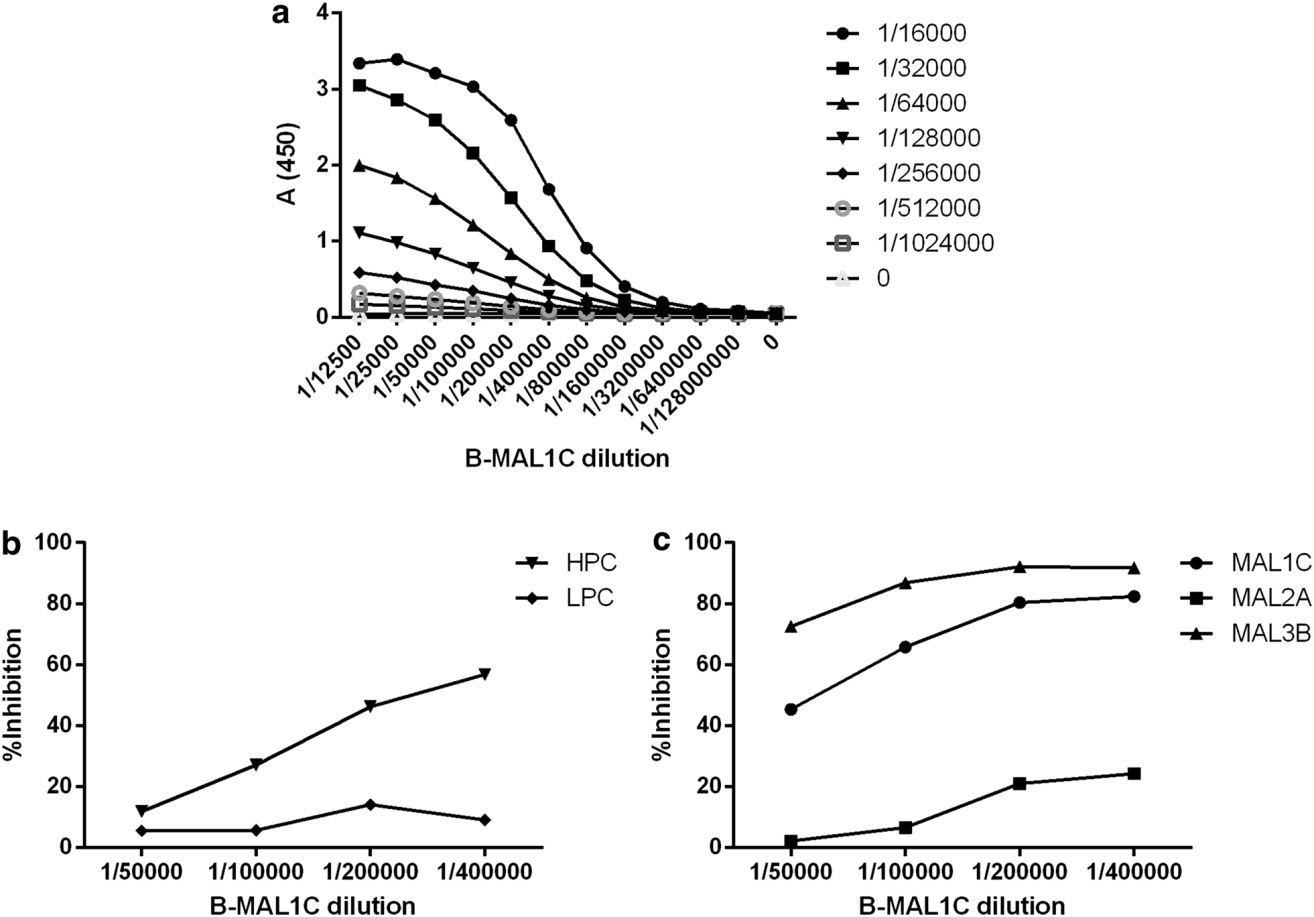
Development of the MAL1C-competition ELISA. Optimal dilutions of B-MAL1C and streptavidin-HRP were defined using a checkerboard titration experiment (**a**). At a 1/16,000 dilution of streptavidin-HRP a sigmoidal curve was observed; this dilution was used for further experiments. B-MAL1C dilutions in the range between the maximal absorbance (plateau observed at 1/50,000) and half max (max/2 observed at 1/400,000) were explored in an inhibition set up using defined sera with high and low antibody content in R32LR ELISA assay (**b**) and the three monoclonal antibodies, MAL1C, MAL2A and MAL3B (**c**). The data shown in *panels b* and *c* were obtained with the highest serum concentrations (starting dilution 1/5) and with mAb concentrations of 5 µg/ml

Plates were coated with the capturing antigen His-R32LR as described in “Methods” section. Optimal dilutions of B-MAL1C and streptavidin-HRP were determined using a checkerboard titration set-up. The streptavidin-HRP conjugate dilutions ranged between 1/16,000 and 1/1,024,000 and B-MAL1C dilutions between 1/12,500 and 1/12,800,000. A prototypic sigmoidal curve was obtained at a streptavidin-HRP conjugate concentration of 1/16,000 (Fig. 1a). This dilution was, therefore, used in further experiments. An optimal B-MAL1C dilution was chosen in the range that produced between 50 and 100% of the maximal absorbance (*A*_450_) value, which was between 1/50,000 (100%) and 1/400,000 (50%). To find the most discriminating assay condition, dilutions of B-MAL1C (1/50,000, 1/100,000, 1/200,000 and 1/400,000) were tested in a competition ELISA set-up with a low positive control (LPC) and high positive control (HPC) serum (Fig. 1b) and non-labelled monoclonal antibodies MAL1C, MAL2A, MAL3B (5 µg/ml starting dilution) (Fig. 1c). Serial twofold dilutions HPC, LPC and mAbs were examined in an inhibition set-up. The maximum inhibition and most discriminative results HPC and LPC were observed in the least diluted sample (starting dilution 1/5) and at a 1/400,000 dilution of B-MAL1C. At this dilution of B-MAL1C also maximal inhibitory effects of 5 µg/ml of the three mAbs were noted (Fig. 1c). Therefore, a 1/400,000 dilution of B-MAL1C was used in further testing and assay evaluation. The degree with which polyclonal sera or mAbs inhibited the interaction of B-MAL1C with the R32LR capturing antigen was expressed as % inhibition relative to a non-inhibiting control sample as described in “Methods” section.

### Competition ELISA with MAL1C, MAL2A and MAL3B

Figure 1c further demonstrates that the three mAbs (MAL1C, MAL2A, MAL3B) differently inhibit the interaction of B-MAL1C (at optimal dilution of 1/400,000) with the R32LR capturing antigen. MAL3B displays the strongest inhibition not only when mixed with the B-MAL1C diluted at 1/400,000 but also when mixed with higher amounts of B-MAL1C (1/50,000–1/200,000). MAL1C behaves largely like MAL3B but MAL2A exhibited much lower inhibitory activity than the two other mAbs.

**Fig. 2:**
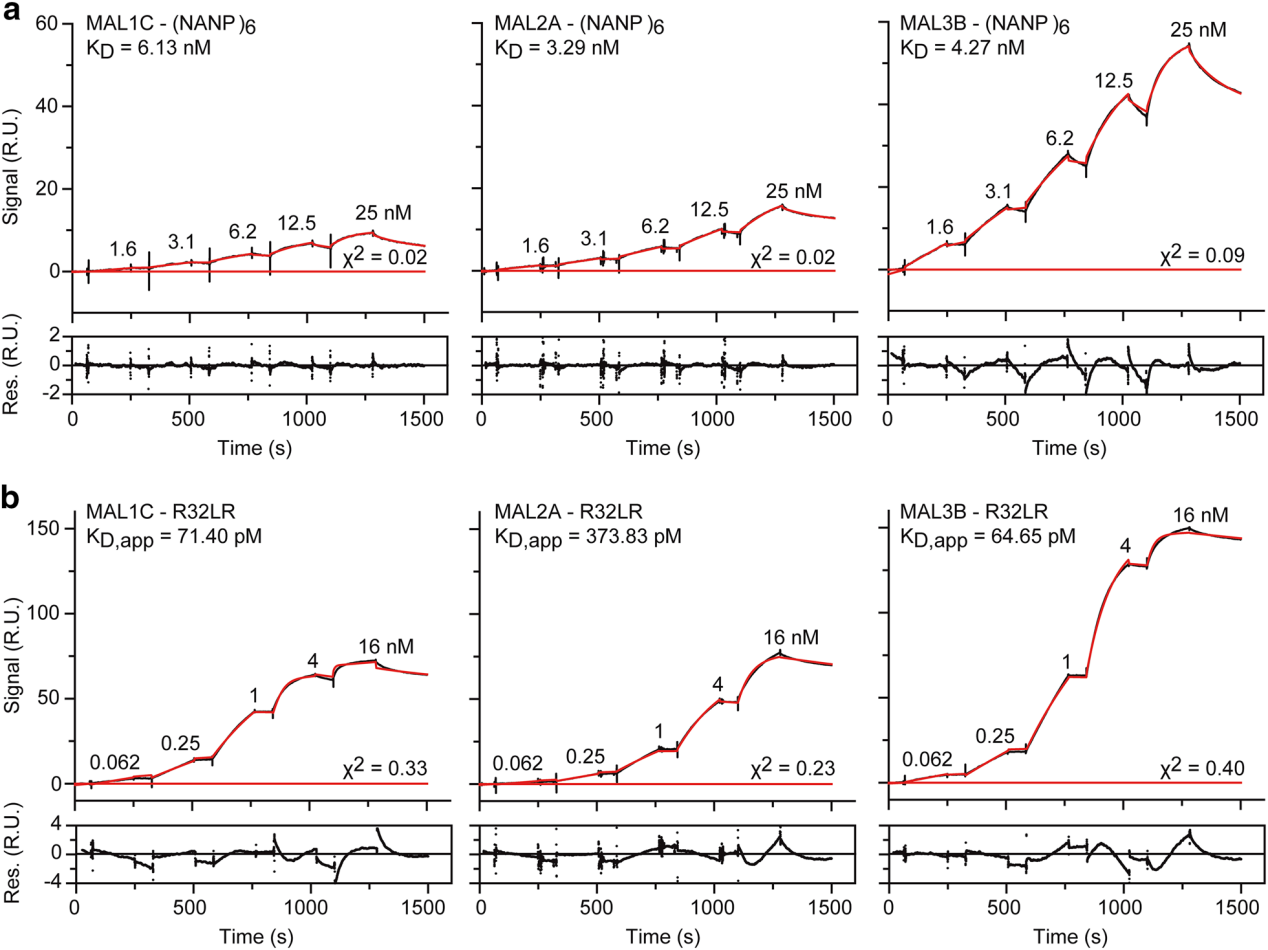
Interactions between the three mAbs (MAL1C, MAL2A, and MAL3B) and the CSP-derived peptides as measured by SPR. The data for the mAb:CSP-peptide interaction were measured in the format of a kinetic titration [30], with mAb as the ligand (i.e., coated onto the sensor surface) and either (NANP)_6_ (**a**) and R32LR (**b**) as the analyte. In both panels, the top *graphs* display the sensorgrams (*black traces*) and the fit to the data with a 1:1 Langmuir binding model (*red traces*). The residuals of the fit are shown in the bottom *graph*. The (apparent) affinity constants for the interactions and the chi2 of the fit are also shown for convenience. *K*_*D*_ dissociation constant, *K*_*D, app*_ apparent dissociation constant, *s* seconds, *RU* resonance units

To explore these differences further, the binding interactions between the mAbs (MAL1C, MAL2A, MAL3B) and the CSP-derived proteins ([NANP]_6_ and R32LR) were analysed via surface plasmon resonance (SPR). In the experimental set-up, the mAbs were coated onto the sensor surface, whereas the CSP-derived peptides were selected as analytes. First, the binding of the three mAbs to (NANP)_6_ was evaluated. As can be seen from Fig. 2 and Additional file 1: Table S1, the three mAbs display no difference in (NANP)_6_-binding behaviour as MAL1C, MAL2A, and MAL3B possess similar affinities in the nM range. However, upon evaluation of mAb binding to R32LR, a significant difference could be observed between MAL1C and MAL3B on one hand, and MAL2A on the other. While MAL1C and MAL3B interact with R32LR with very comparable apparent affinities, MAL2A binding to R32LR is fivefold weaker (Fig. 2 and Additional file 1: Table S1).

### Correlation between MAL1C-competition ELISA and R32LR ELISA

Antibody concentrations were measured in sera from 46 participants from study 1 obtained at three points in time (before vaccination, 4 weeks after the 2nd dose (D56) and 3 weeks after the administration of the 3rd vaccine dose). Similarly, antibody contents were also measured in sera from 46 participants from study 2. The samples were taken prior to vaccination in all participants and 4 weeks after the second dose (D56) and 21 days after the 3rd dose (D217) in one study group and prior to the 3rd dose (D196, or 168 days after the 2nd dose), and 21 days after the 3rd dose (D217) in the other group (Additional file 2: Figure S1). All sera were analysed with both the R32LR ELISA and the MAL1C-competition ELISA. In the latter, optimal reagent conditions (1/16,000 streptavidin-HRP conjugate dilution and 1/400,000 B-MAL1 dilution) were applied. Prior to vaccination no antibodies could be detected with either method.

**Fig. 3:**
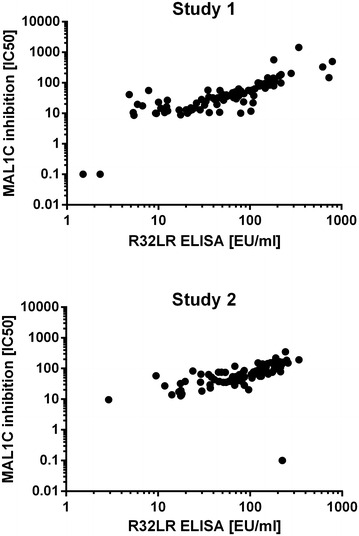
Correlation between R32LR ELISA and MAL1C-competition ELISA. Sera from participants in study 1 (*panel A*) and study 2 (*panel B*) taken after the second and third vaccine doses (immediately prior to mosquito bite challenge) were analysed with the standard R32LR ELISA and the MAL1C-competition ELISA. Results obtained with both assays are plotted and correlation is calculated with Pearson’s correlation

Using the assay developed herein, MAL1C-like antibodies were detected in most post-vaccination sera. Figure 3 shows an overall good correlation between the results obtained in both assays. The Pearson correlation coefficients were 0.705 (P < 0.0001) and 0.5461 (P < 0.0001) in the study 1 and 2 sample sets, respectively.

**Fig. 4:**
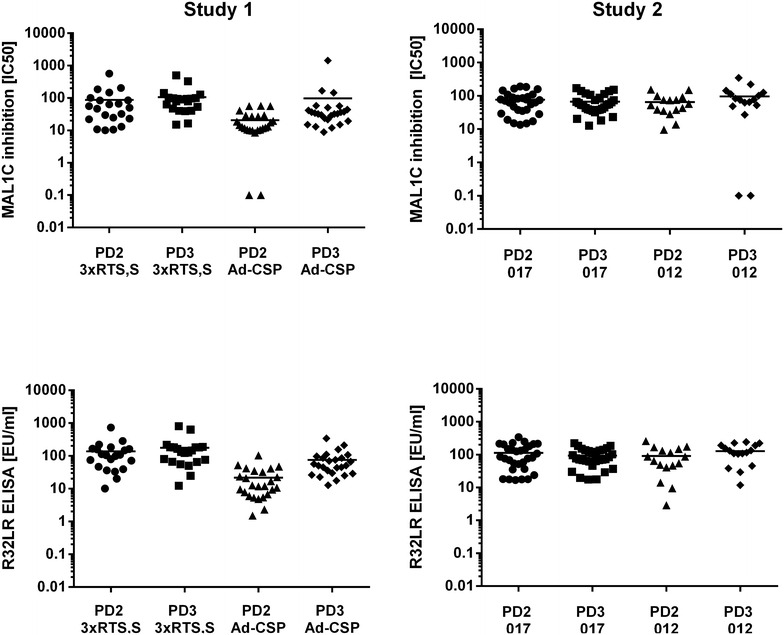
Comparison of antibody concentrations in sera from different time points and treatment groups. In both studies sera were obtained after the second (PD2) and third (PD3) vaccine dose and antibody concentrations were measured with R32LR and MAL1C-competition ELISA. In study 1 results obtained in the subjects receiving three doses of RTS,S/AS01 (3xRTS,S/AS01) were compared with those generated in subjects vaccinated with one dose of Ad35.CS.01 followed by two doses of RTS,S/AS01. In study 2, subjects vaccinated with three standard doses of RTS,S/AS01 given with monthly intervals at month 0, 1 and 2 (012) were compared with subjects given a standard dose at months 0 and 1 and 1/5 th of the standard dose at month 5 (017). No significant differences were observed between the study groups or between 2 and 3 vaccine doses

The antibody levels measured with both assays at different time points, elicited by varying vaccination schemes in both studies allowed a comparison of the MAL1C-like antibody profiles with those generated with the anti-CS ELISA. Figure 4 shows that both assays generate data displaying similar patterns and that the measurement of MAL1C-like antibodies does not provide additional information. Antibody levels measured after three doses of RTS,S/AS01were not significantly higher than those measured after two doses. Post dose 3, different vaccine regimens in study 1 and administration schemes in study 2 had no significant impact on antibody levels, irrespective of the assay used.

**Fig. 5:**
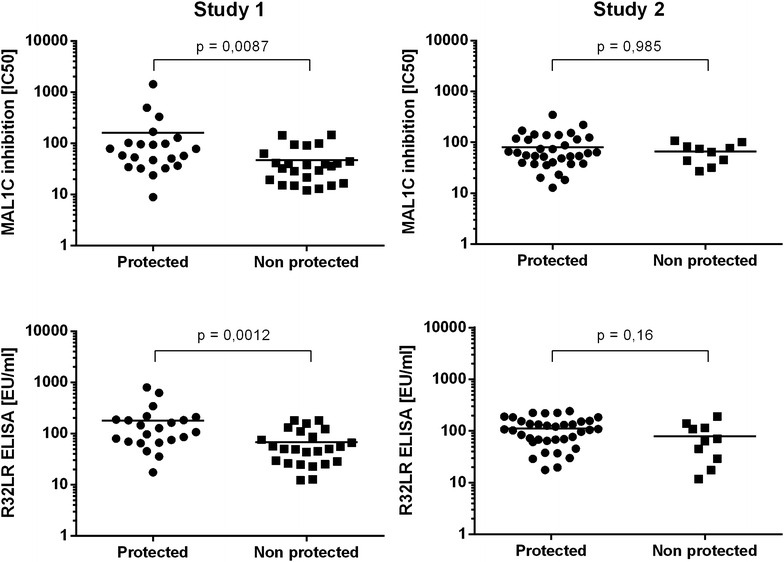
Correlation between protection from infection and antibody content, measured with R32LR and MAL1C-competition ELISA. Antibody concentrations in the sera obtained immediately before challenge with infected mosquito’s from participants at the study 1 (n = 46) and study 2 (n = 46) studies were measured with the R32LR ELISA and MAL1C-competition ELISA. Antibody levels in protected and non-protected vaccine recipients were compared and no significant differences were observed irrespective of the assay used

Finally, it was examined whether the antibody content measured with the MAL1C-competition ELISA would reveal a better association with protection from infection than that measured with the R32LR ELISA. In samples from both studies and irrespective of the assay method used, no association was observed between the antibody content and the protection status of the vaccine recipients (Fig. 5). The MAL1C-competition ELISA, which quantifies only the MAL1C-like antibodies in a polyclonal serum sample, provides no additional information about the protective capacity of polyclonal sera.

## Discussion

A competitive ELISA was developed to measure in sera from RTS,S/AS01 vaccine recipients the fraction of polyclonal antibodies that compete with MAL1C, a human monoclonal antibody, derived from an RTS,S/AS01 vaccinated volunteer, directed against the central repeat region of CSP. A survey of 276 sera derived from 92 participants in two clinical trials that examined the protective efficacy of the RTS,S/AS01 candidate vaccine, demonstrated that most, if not all, of the polyclonal antibodies present in sera of study participants behaved like the human monoclonal antibody MAL1C, suggesting they compete for the same binding site(s) on the capturing antigen, R32LR.

Polyclonal antibodies elicited by vaccination with RTS,S/AS01 and directed against the central repeat region of the CSP are able to protect vaccine recipients against challenges with infected mosquitoes [13, 31–33]. Administration of human mAbs directed to the central repeat region of CSP (MAL1C, MAL2A, MAL3B), derived from one RTS,S/AS01 vaccine recipient, was also shown to convey protection against *P. falciparum* infection in immune deficient mice with human liver tissue [23]. Although no precise antibody threshold has been defined above which protection from infection is secured, some association between anti-CS concentration and protection from infection has been observed in man [13, 18–22] and mice [23]. However the overlap in antibody concentrations of protected and non-protected individuals as measured with the standard R32LR ELISA is considerable. The use of the MAL1C-competition ELISA compared to the R32LR ELISA does not provide additional information or discriminatory power.

The protective role of antibodies directed against the central repeat region of CSP is well established. However the inability to define a precise correlate of protection and the large overlap of anti-CS concentrations between protected and unprotected populations suggest that other factors may be involved. Apart from the actions of cells from the innate (NK, NKT) and adaptive immune system (CD4, CD8), also less explored antibody qualities such as affinity/avidity or antibody specificities that are not examined with the R32LR ELISA assay may contribute to protection.

Olotu et al. [34] have examined the avidity of anti-circumsporozoite antibodies, as measured using an elution ELISA method, in RTS,S/AS01_E_-vaccinated children residing in malaria endemic countries. In this survey no association between antibody avidity and protection from clinical malaria has been observed.

The different behaviour of the three mAbs (MAL1C, MAL2A and MAL3B) in the MAL1C-competition ELISA (Fig. 1b) led to study their binding interactions with the CSP-derived peptides ([NANP]_6_ and R32LR) via surface plasmon resonance (SPR). While the three mAbs showed a similar (NANP)_6_-binding behaviour, MAL2A bound fivefold weaker to R32LR than MAL1C and MAL3B, which displayed very comparable apparent affinities. Despite these observed differences in SPR and competition ELISA, the three mAbs display an equal protective capacity in the humanized uPA-SCID challenge model [23]. All three mAbs are extremely good binders of CSP with apparent picomolar affinities, which explains the in vivo protective effect of the mAbs when administered separately to mice with human livers at a dose of 400 µg/animal. However, when placed in direct competition to each other, differences can be observed between the three mAbs. Indeed, MAL1C and MAL3B have better apparent affinities than MAL2A, which is why they score better in an inhibition assay.

The ELISA assays employed here only detect antibodies recognizing the central repeat region of CSP and do not provide information on antibodies directed against the carboxy-terminal part of the RTS,S/AS01 vaccine antigen. Data about the amount of such antibodies that are induced by RTS,S/AS01 vaccination and their possible role in protection is scarce. In study 2, antibody titers against the C-terminus were shown not to be associated with protection [26]. Sera from malaria-naïve human volunteers infected with sporozoites under chloroquine prophylaxis (CPS vaccination) have protective qualities despite their low levels of antibodies directed against the central repeat region of CSP [35]. Antibodies that remain undetected with the R32LR ELISA may be responsible for this protection and deserve further exploration.

## Conclusion

A competition ELISA to measure MAL1C-like antibodies in polyclonal sera from RTS,S/AS01 vaccine recipients has been developed and showed that MAL1C-like antibodies that recognize the same epitope as the mAb MAL1C are induced by RTS,S/AS01 vaccination. An excellent correlation was observed between the results generated with the MAL1C-competition ELISA and those generated with the validated standard ELISA. In contrast to the findings in mice where protection from infection was directly associated with circulating MAL1C mAb concentrations, no such association was observed between, MAL1C-like antibody concentrations detected in sera from RTS,S/AS01 vaccine recipients and protection from infection following infected mosquito bites. These results warrant further in depth analysis of the composition of polyclonal sera from RTS,S/AS01 vaccine recipients and further assessment of the binding qualities and function of these antibodies.

## Additional files

**Additional file 1: Table S1.** Kinetic parameters of the mAb:CSP-peptide interaction.

**Additional file 2: Figure S1.** Overview of the design of the two clinical vaccine studies from which the serum samples have been derived.
