# Supplementary material for: A monoclonal antibody-based immunoassay to measure the antibody response against the repeat region of the circumsporozoite protein of Plasmodium falciparum
Source: Malar J. 2016 Nov 8;15:543. doi: 10.1186/s12936-016-1596-8 (PMC5101676; doi:10.1186/s12936-016-1596-8)
Supplement: Supplementary file 1 — Additional file 1: Table S1. Kinetic parameters of the mAb:CSP-peptide interaction. [file 12936_2016_1596_MOESM1_ESM.docx]

Additional Table S1

**Kinetic parameters of the mAb:CSP-peptide interaction.**

|  | **R32LR** | | | **(NANP)_6_** | | |
| --- | --- | --- | --- | --- | --- | --- |
| **mAb** | **k_on_**  **[10^+5^ M^−1^ s^−1^]** | **k_off_**  **[10^−3^ s^−1^]** | **K_D,app_**  **[pM]** | **k_on_**  **[10^+5^ M^−1^ s^−1^]** | **k_off_**  **[10^−3^ s^−1^]** | **K_D_**  **[nM]** |
| **MAL1C** | 88.20 | 0.63 | 71.40 | 10.50 | 6.44 | 6.13 |
| **MAL2A** | 10.70 | 0.40 | 373.83 | 37.40 | 12.30 | 3.29 |
| **MAL3B** | 23.20 | 0.15 | 64.65 | 11.30 | 4.83 | 4.27 |

k_on_ = association rate; k_off_ = dissociation rate; K_D_ = affinity constant, K_D,app_ = apparent affinity constant.
