# Supplementary material for: A monoclonal antibody-based immunoassay to measure the antibody response against the repeat region of the circumsporozoite protein of Plasmodium falciparum
Source: Malar J. 2016 Nov 8;15:543. doi: 10.1186/s12936-016-1596-8 (PMC5101676; doi:10.1186/s12936-016-1596-8)

**Additional File 2: Figure S1**

**Overview of the design of the two clinical vaccine studies from which the serum samples have been derived**. Study 1 (described in detail (25)) examined the safety, immunogenicity and protective efficacy of two vaccine regimens applying the same vaccination administration schedule (0, 1, 2 months) but using either three doses of the RTS,S vaccine (RRR Group) or one dose of the Ad35.CS.01, a replication deficient adenovirus type 35 circumsporozoite malaria vaccine (dose 1) followed by two doses of RTS,S vaccine (ARR Group).Three weeks after the last (3^rd^) vaccine dose participants where challenged by a controlled human malaria parasite infection (CHMI), through the bite of 5 *P. falciparum* infected *Anopheles stephensi* mosquitoes. Study 2 (described in detail (26)) examined the safety, immunogenicity and protective efficacy of RTS,S/AS01_B_ administered intramuscularly as standard doses at 0 and 1 month and a 1/5^th^ standard dose at 7 months (delayed fractional dose group, 017 Group) as compared to RTS,S/AS01_B_ administered as three standard doses at monthly intervals (0, 1, 2 months group, 012 Group) Vaccine doses are depicted as black squares on the time line and represented by R for a standard dose of RTS,S, A for a dose of Ad35.CS.01 and r for a reduced (1/5 of standard dose) of RTS,S. CHMI is shown as a black diamond and blood samplings by arrows. Number of vaccine recipients in each study group are shown as well as the number of subjects challenged and the number protected.


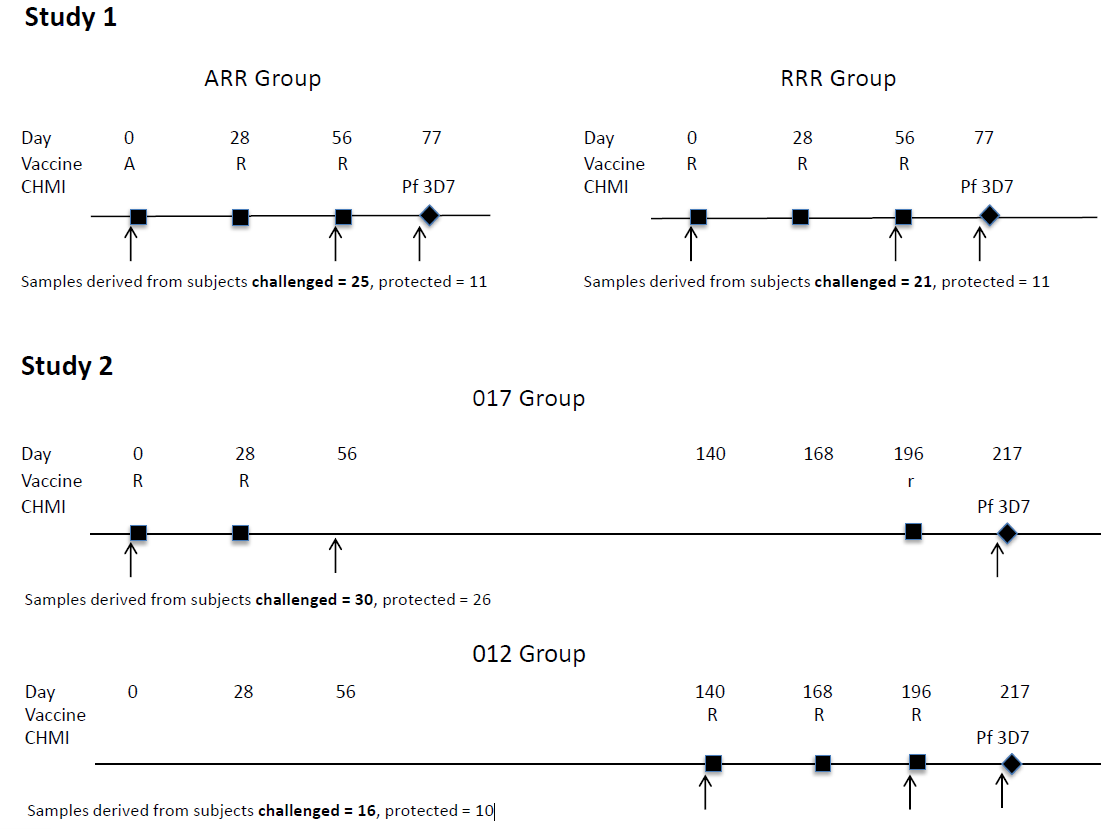

Supplement: Supplementary file 2 — Additional file 2: Figure S1. Overview of the design of the two clinical vaccine studies from which the serum samples have been derived. [file 12936_2016_1596_MOESM2_ESM.docx]
